# The Effect and Mechanism of Corilagin from *Euryale Ferox* Salisb Shell on LPS-Induced Inflammation in Raw264.7 Cells

**DOI:** 10.3390/foods12050979

**Published:** 2023-02-25

**Authors:** Minrui Wu, Yuhan Jiang, Junnan Wang, Ting Luo, Yang Yi, Hongxun Wang, Limei Wang

**Affiliations:** 1 College of Life Science and Technology, Wuhan Polytechnic University, Wuhan 430023, China; 2 College of Food Science and Engineering, Wuhan Polytechnic University, Wuhan 430023, China

**Keywords:** inflammatory reaction, Raw264.7 macrophage, corilagin from *Euryale ferox* Salisb shell, cell pathway

## Abstract

(1) Background: *Euryale ferox* Salisb is a large aquatic plant of the water lily family and an edible economic crop with medicinal value. The annual output of *Euryale ferox* Salisb shell in China is higher than 1000 tons, often as waste or used as fuel, resulting in waste of resources and environmental pollution. We isolated and identified the corilagin monomer from *Euryale ferox* Salisb shell and discovered its potential anti-inflammatory effects. This study aimed to investigate the anti-inflammatory effect of corilagin isolated from *Euryale ferox* Salisb shell. (2) Methods: We predict the anti-inflammatory mechanism by pharmacology. LPS was added to 264.7 cell medium to induce an inflammatory state, and the safe action range of corilagin was screened using CCK-8. The Griess method was used to determine NO content. The presence of TNF-α, IL-6, IL-1β, and IL-10 was determined by ELISA to evaluate the effect of corilagin on the secretion of inflammatory factors, while that of reactive oxygen species was detected by flow cytometry. The gene expression levels of TNF-α, IL-6, COX-2, and iNOS were determined using qRT-PCR. qRT-PCR and Western blot were used to detect the mRNA and expression of target genes in the network pharmacologic prediction pathway. (3) Results: Network pharmacology analysis revealed that the anti-inflammatory effect of corilagin may be related to MAPK and TOLL-like receptor signaling pathways. The results demonstrated the presence of an anti-inflammatory effect, as indicated by the reduction in the level of NO, TNF-α, IL-6, IL-1β, IL-10, and ROS in Raw264.7 cells induced by LPS. The results suggest that corilagin reduced the expression of TNF-α, IL-6, COX-2, and iNOS genes in Raw264.7 cells induced by LPS. The downregulation of the phosphorylation of IκB-α protein related to the toll-like receptor signaling pathway and upregulation of the phosphorylation of key proteins in the MAPK signaling pathway, P65 and JNK, resulted in reduced tolerance toward lipopolysaccharide, allowing for the exertion of the immune response. (4) Conclusions: The results demonstrate the significant anti-inflammatory effect of corilagin from *Euryale ferox* Salisb shell. This compound regulates the tolerance state of macrophages toward lipopolysaccharide through the NF-κB signaling pathway and plays an immunoregulatory role. The compound also regulates the expression of iNOS through the MAPK signaling pathway, thereby alleviating the cell damage caused by excessive NO release.

## 1. Introduction

The immune stimulator, LPS, can activate multiple signaling pathways in macrophages, leading to a series of pathophysiological responses [1], and is often used in in vitro inflammation studies. Inhibition of excessive activation of macrophages and its mediated inflammation has been demonstrated to be beneficial in many disease models [2,3], suggesting that targeting macrophage activation is a promising strategy for preventing inflammatory diseases.

*Euryale ferox* Salisb is a large aquatic plant belonging to the water lily family, which has been frequently reported for its use in lowering blood sugar and blood lipid [4], in addition to its antioxidation properties [5]. Shuliang He et al. characterized the constituents of the volatile oil of *Euryale ferox* Salisb and identified its biological activity, particularly its antioxidant activity [6]. Additionally, Wen-Na Zhang et al. characterized the polysaccharide in *Euryale ferox* Salisb and investigated its hypoglycemic effect [7]. The biosynthesis mechanism of flavonoids in *Euryale ferox* Salisb was analyzed by Peng Wu et al. through metabolomic and transcriptomic analyses, which revealed the key factors involved in the biosynthesis of flavonoids in *Euryale ferox* Salisb, its main functional substances [8]. Transcriptomic analysis of *Euryale ferox* Salisb at different developmental stages was performed by Xian Liu et al. [9], knowledge of which is particularly useful for the development and utilization of *Euryale ferox* Salisb.

With an annual output of more than 1000 tons, the *Euryale ferox* Salisb shell accounts for around 40% of the seed. It is often used as fuel or transported for disposal, resulting in waste of resources and environmental pollution [10]. Cheng Ying Wu et al. studied the antioxidant and anti-fatigue properties of phenolic extracts of the *Euryale ferox* Salisb shell, which led to the discovery of potential antioxidant agents [11].

Corilagin has been reported to exhibit various pharmacological activities, including inhibition of inflammatory development [12], antiviral [13], liver protection [14], and antitumor effects [15]. Li et al. [16] found that corilagin significantly reduced the levels of IL-6 and IL-1β in the serum of cells and mice and exhibited an anti-inflammatory role by downregulating the TLR4 signaling molecules, improving the extreme inflammatory state in patients with sepsis. Additionally, Tong et al. [17] found that corilagin may inhibit the activation of the nuclear factor-κB pathway in a STAT3-related manner and reduce the secretion of IL-1β and TNF-α, thereby reducing radiation-induced brain injury in mice. Previous studies have demonstrated that corilagin mainly improves cellular inflammation through the TLR signaling pathway, but there is no report of its activity in the LPS-induced RAW264. Previously, we isolated and identified the corilagin monomer from *Euryale ferox* Salisb shell; however, the anti-inflammatory effect was not evaluated.

We applied a network pharmacology approach to predicting the anti-inflammatory mechanism of corilagin, using the LPS-stimulated Raw264.7 cells as an in vitro inflammatory model to determine the effect of corilagin from *Euryale ferox* Salisb on the expression of inflammatory and anti-inflammatory factors in macrophages and the possible molecular mechanisms at three levels, i.e., biochemical factors, transcription, and protein levels. The theoretical groundwork is provided by developing and utilizing the Euryale ferox Salisb shell.

## 2. Materials and Methods

### 2.1. Materials and Chemicals

Raw264.7 cells were purchased from Shanghai Cell Bank, Chinese Academy of Sciences. The cell viability detection kit was purchased from Japan Tongren Reagent Company, and lipopolysaccharide for inducing inflammation was purchased from Sigma Company. The ELISA kit was purchased from Beijing Sizheng Bo Bio Co., Ltd. (Beijing, China), the ROS kit was purchased from Beijing Prilai Gene Technology Co., Ltd. (Beijing, China), and the reverse transcription kit was provided by Bao Bioengineering Co., Ltd. (Dalian, China). Primers were provided by Shanghai Sangon Biology Co., Ltd. (Shanghai, China). The primary and secondary antibodies used for Western blotting were provided by CST (Pi3K and p-Pi3K, Bioss; AKT and JNK, Wuhan Miting).

### 2.2. Separation and Identification of Corilagin from Euryale ferox Salisb Shell

*Euryale ferox* Salisb shells were dried and crushed, filtered with a 200-mesh sieve, ultrasonically extracted with 70% ethanol, concentrated under reduced pressure, and freeze-dried to obtain *Euryale ferox* Salisb shell polyphenol alcohol extracts. The chitosan polyphenol extract was added with water and ultrasonicated before being fractionally extracted with petroleum ether, ethyl acetate, and n-butanol (*v*/*v* = 1:1). The extract phase of the *Euryale ferox* Salisb shell was collected and packed on a silica gel column (60 mesh) by the wet method for separation. Elution was carried out with a mixture of ethyl acetate and petroleum ether (2:1), and the elution fractions were collected. The fraction with the highest activity was concentrated and lyophilized. The eluent was petroleum ether:ethyl acetate (100:15), and the eluent was collected. The samples were separated on a Sephadex LH-20 (Hydroxypropyl Sephadex) chromatographic column with 50% methanol and water, and the monomer compound was obtained by semi-preparative liquid phase, which was identified as corilagin (Figures S1 and S2).

### 2.3. Corilagin and Inflammation Target Prediction and Screening Application

The 2D structure of corilagin was obtained from the PubChem database, and the sdf file of the drug structure was imported into PharmMapper and Swiss Target Prediction, the TCMSP database, to obtain drug-related targets by merging and de-weighting. The disease-related targets were obtained from the GeneCards database by setting the search keyword “inflammation” as the genus “human origin”.

### 2.4. Construction of PPI Network and Acquisition of Crossover Genes

The species was set as the human species, and the minimum relationship score was 0.4. The key proteins with the cross-repetition of corilagin and inflammation were input into the protein interaction database (STRING), and the proteins without an interaction relationship were removed to obtain the protein interaction map.

### 2.5. HUB Genes’ Acquisition and KEGG and GO Enrichment Analysis

Cytoscape 3.9.1 was supplied with the protein–protein interaction diagram obtained from the STRING database to obtain the top 30 central target genes for interactions with other proteins in the network diagram. GO and KEGG analysis of central target genes were performed using the R-package clusterProfiler and enrichment plot. The data with *p*-value < 0.05 were screened, and the relevant legends were plotted using the R package ggplot2.

### 2.6. Docking Analysis

The top five degrees in the PPI network were used as the receptor proteins, the top nodes in the “active ingredient-target-disease” network were used as the ligands, and the structures of the receptor proteins were downloaded from the PDB database. The proteins and ligands were pre-processed using PyMOL-2.3.4. Subsequently, AutoDockTools software was used to pre-process the protein and ligands, and Vina was used for predicting the binding energy of the ligands of small size to the proteins, with the lowest binding energy indicating the optimal conformation. The receptor–ligand docking files were processed by PyMOL and uploaded to the online website called Plip to visualize the validation results.

### 2.7. Cell Culture and Model Establishment

Raw264.7 cells, the mouse monocytic leukemia cells, were cultured in a DMEM medium containing 10% FBS and 1% penicillin and streptomycin dual-antibodies at 37 °C in an incubator containing 5% CO_2_. The culture was passaged when grown to more than 90% in cell culture flasks, and selected experiments were performed on counted cells. The experiments were conducted by comparing different groups of experimental subjects: the control group (without LPS and corilagin intervention), the LPS stimulation group (with the addition of 1 μg/mL of LPS for intervention), the experimental group (different concentrations of corilagin were pretreated for 2 h and the final concentration of 1 μg/mL was added and LPS co-treated for 24 h), and the positive drug group (50 μmol/L of dexamethasone pretreatment for 2 h, LPS with a final concentration of 1 μg/mL added for 24 h).

### 2.8. Cell Morphology Observation and CCK-8 Assay to Detect the Proliferation Toxicity of Corilagin on Raw264.7 Cells

Cells were seeded into 6-well plates (at a density of 5 × 10^5^ cells per well). The cells were divided into a normal control group, an LPS stimulation group, and a corilagin treatment group, and the experiments were conducted in replicates (2 wells for each group). The cellular morphology was observed by an inverted microscope.

To screen for the safe concentration of corilagin from the Gorgon husk source, the CCK-8 method was used to analyze the effect of corilagin on the survival rate of Raw264.7 cells.

Cells in the logarithmic growth phase were sampled for cell counting, and 100 μL of cell suspension was added to each well of a 96-well plate (density of 3000 cells per well). The surrounding wells were sealed with PBS and grown for 24 h.

The experiment was divided into the blank group (without cells and drugs), the control group (without drugs), and the experimental group. The drugs were prepared by diluting with complete medium to 2-fold gradient dilution, followed by filtration with a membrane of 0.22 μm pore size, and used immediately. The cultures were grown for 24 h before the analysis. When testing, the medium in the 96-well plate was aspirated, washed twice with PBS, and patted dry on thick paper. CCK-8 was prepared in the dark to avoid errors caused by residual CCK-8 in the pipette tip left when adding samples. A complete medium was used to dilute CCK-8, and the diluted solution was mixed well for later use. The cultures were incubated for 3 h in an incubator, and the OD value at a wavelength of 450 nm was determined.

### 2.9. Determination of NO Content by Griess Method

Raw264.7 cells were seeded into a 24-well plate (at a density of 2 × 10^5^ cells per well) and placed in a cell incubator for 12 h. Different groups were cultured for 24 h according to the corresponding treatment, and the cell supernatant was collected. The NO content in the supernatant was detected by the Griess reagent method, and the amount of released NO of each group was calculated using the standard curve.

### 2.10. ELISA Method to Determine the Effect of Corilagin on the Secretion of Inflammatory Factors

Cell treatment was kept consistent with the pretreatment method used for cell morphology observation, and the cell supernatant was collected. The contents of TNF-α, IL-6, IL-1β, and IL-10 were determined according to the instructions of the ELISA kit.

### 2.11. Detection of Intracellular Reactive Oxygen Species by Flow Cytometry

The cells in the logarithmic growth phase were inoculated into 6-well plates and cultured for 12 h. Different groups were cultured for 24 h according to the corresponding treatment. The liquid in the 6-well plate was discarded and washed twice with PBS. The control group was added with 2 mL of complete medium. The base, lipopolysaccharide, and experimental groups were added with 2 mL of 20 μmol DCFH-DA diluted in a complete medium and incubated in an incubator for 2 h. After incubation, PBS was used for rinsing twice, i.e., 1 mL of PBS was added to each well and the cells were detached by pipetting, collected into a centrifuge tube, and centrifuged at 1000 r/min for 3 min, the supernatant was discarded, and 1 mL of PBS was added to each tube. The mixtures were mixed by pipetting, the cell suspension was transferred to a 1.5 mL flow centrifuge tube, and the intensity of the intracellular ROS fluorescence was measured by flow cytometry.

### 2.12. qRT-PCR Detection of TNF-α, IL-6, COX-2, and iNOS Gene Expression Levels

The six-well plate was taken out, and the medium discarded and rinsed twice with PBS. Then, 1 mL of RNA iso plus was added and left to stand for 1 min before pipetting. The cell suspension was collected by pipetting, transferred into a sterile tube, and added with 200 μL of chloroform, and the mixture was mixed well. Next, extraction was carried out on the ice for 15 min with inversion every 5 min. The EP tube with three layers of supernatant was carefully removed, and the supernatant was pipetted into another 1.5 mL EP tube using a 100 μL pipette. Then, 500 μL of isopropanol was added and mixed well, and the tube was placed on ice for 15 min. The mixture was then centrifuged at 12,000 rpm and at 4 °C for 15 min to recover a white precipitate. The supernatant was carefully removed to avoid disturbing the pellet. Next, 1 mL of 75% ethanol was added, and the pellet was gently lifted. The wall of the tube was washed by inversion, at 7500 rpm. The supernatant was discarded, and the pellet was air-dried with the lid opened at room temperature for 5 min before the addition of an appropriate amount of DEPC water to dissolve the pellet. The RNA concentration was measured using a UV micropipette and adjusted to obtain the RNA concentration of 1000 ng/μL per tube. The RNA was reverse-transcribed into cDNA according to the protocols for reverse transcription, and the real-time fluorescence quantitative PCR reaction system using 10 μL of SYBR Premix Ex Taq TM, 8 μL of primers, and 2 μL of cDNA was conducted.

Primer sequences (Table 1): The cDNA sequences of each gene were retrieved from NCBI, and specific primer sequences were designed and synthesized by Shanghai Sangon Bioengineering Co., Ltd.

### 2.13. Western Blot Analysis of Key Proteins’ Expression in NF-κB, MAPK, and PI3K-AKT Signaling Pathways

After preconditioning cells, the excess medium in the well plate was aspirated, and 1 mL of PBS was used for washing twice. Cells were digested with 1 mL of trypsin, transferred to a 1.5 mL EP tube, and centrifuged at 3000 rpm for 1 min. The resulting supernatant was discarded. Next, 100 μL of lysis buffer (prepared by RIPA lysis buffer and protease inhibitor 1:100) was added to each tube and evenly pipetted. The cells were lysed on ice for 30 min to ensure complete cell lysis. The cells were then centrifuged at 12,000 rpm for 10 min at 4 °C, and the supernatant was collected to obtain the total protein. A 5× protein loading buffer 4:1 was added to the protein sample, mixed by vortexing, and incubated in a water bath at 95 °C for 10 min. The treated samples were stored in a −20 °C refrigerator for later use. The treated protein samples were transferred to 10% SDS-polyacrylamide gel for electrophoretic separation. The PVDF membrane was placed on the glue and covered with wet filter paper and a sponge. The mounted membrane transfer system was secured with the membrane transfer clip, and the transfer was conducted at 200 mA for 1 h. The membrane was then blocked with 5% skimmed milk at room temperature for 1 h with gentle shaking, followed by overnight incubation with a primary antibody at 4 °C. The blocked membrane was then washed thrice on a decolorizing shaker for 5 min at room temperature. Two hours later, the membrane was then washed thrice on a decolorizing shaker for 5 min. The membrane was then exposed to ECL, and the signal was analyzed using gel imaging software.

### 2.14. Data Statistics and Analysis

GraphPad Prism 8 was used for statistical analysis. All data are expressed as the mean ± standard deviation unless otherwise stated. The *p*-value of <0.05 was considered significant.

## 3. Results

### 3.1. Acquisition of Corilagin and Inflammatory Targets and the “Corilagin-Target-Inflammation” Interaction Network

**Figure 1:**
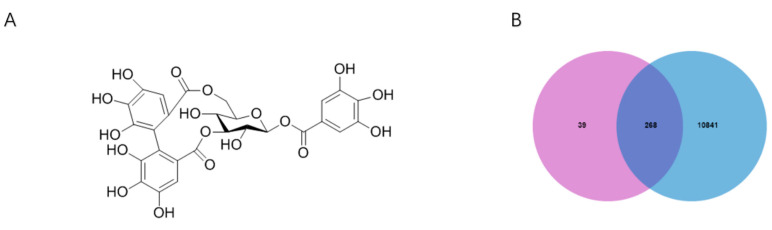
Corilagin and inflammation intersection targets: (**A**) structural formula of corilagin and (**B**) intersection Venn diagram.

**Figure 2:**
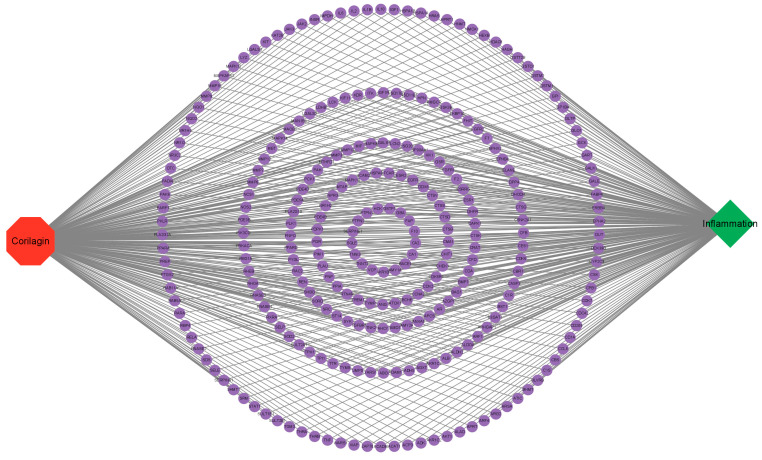
“Corilagin-Target-Inflammation” network.

The 3D structure of corilagin from *Euryale ferox* Salisb shell was obtained from the PubChem database (Figure 1A). A total of 307 corilagin genes were obtained from the PharmMapper website and the Swiss Target Prediction database. Among human species, the GeneCard database showed that there were 111,109 genes related to inflammation. Then, 268 cross-repeating genes (Figure 1B) that appear in both drug targets and inflammatory targets were selected, suggesting that these genes may be the key genes in regulating inflammation of corilagin. To further investigate the relationship between corilagin and inflammation, a “Corilagin-Target-Inflammation” network has been constructed in Cytoscape 3.9.1 (Figure 2).

### 3.2. Construction of PPI Network and Acquisition of HUB Genes

**Figure 3:**
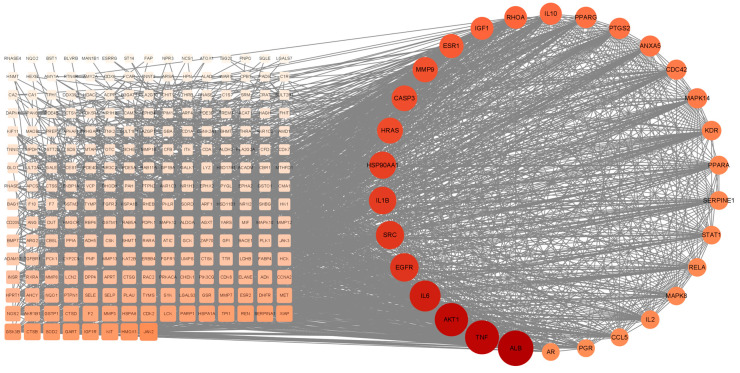
PPI network map and screening of HUB genes.

The crossover genes were imported into the STRING website for protein interactions, and a protein interaction map containing 268 nodes and 3724 edges was obtained. Cytoscape software visualized the protein interaction map, and the top 30 target proteins of the protein interaction network were calculated using the MCC calculation method in the CytoHubba plugin (Figure 3). The results predicted that the above genes and related proteins play an important role in treating hepatocellular carcinoma.

### 3.3. GO and KEGG Pathway Analysis

**Figure 4:**
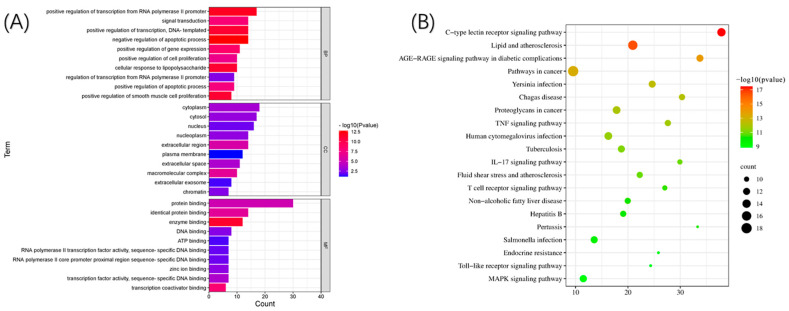
GO and KEGG pathway analyses of HUB genes: (**A**) GO analysis and (**B**) KEGG analysis.

The GO analysis (Figure 4A) revealed the expression of genes localized in the nucleus, while the KEGG (Figure 4B) analysis revealed the MAPK and TOLL-like receptor signaling pathways. Based on findings from the GO and KEGG analyses, it was concluded that corilagin may validate protein expression by regulating the MAPK signaling pathway, which is related to the secretion of inflammatory factors by macrophages, as well as in the NF-B and PI3K signaling pathways, which are closely related to toll-like receptors that attenuate the tolerance level of macrophages.

### 3.4. Validation of Molecular Docking

**Figure 5:**
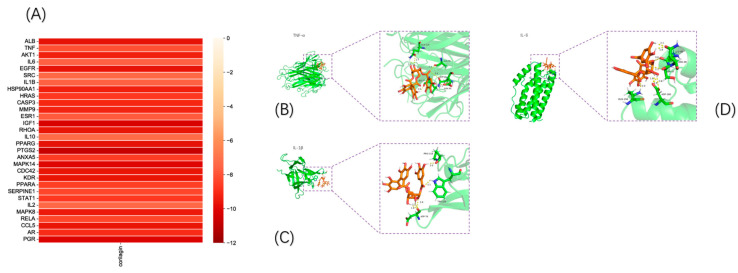
Validation of molecular docking: (**A**) molecular binding energy heatmap and (**B**–**D**) visualization of molecular docking results.

The binding energy (kcal/mol) between the target and corilagin was predicted based on molecular docking (Figure 5A), whereby the negative binding energy of the ligand and receptor usually indicates the binding affinity between them. The docking revealed that the binding energy was less than −5 kcal/mol between all hub gene targets and keratine, from which only three models with good binding energy were selected for visualization (Figure 5B–D).

### 3.5. The Effect of Corilagin on the Viability of Raw264.7 Cells and Changes in Cellular Morphology

**Figure 6:**
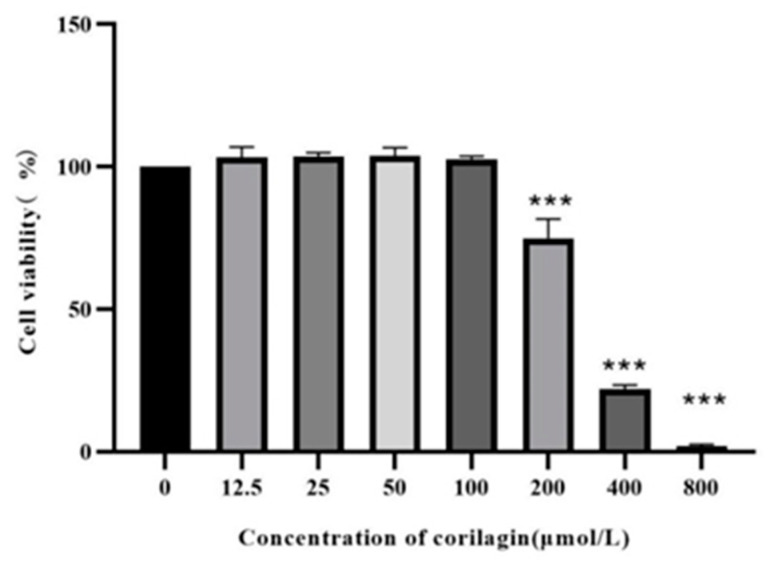
Effect of LPS on Raw264.7 cell viability. (Compared with control group, *** *p* < 0.001).

The investigation of the effect of corilagin on the viability of Raw264.7 cells demonstrated that (Figure 6) cell viability was significantly decreased after incubation for 24 h at concentrations exceeding 100 µm/L, i.e., 25, 50, and 100 μmol/L were selected as the low, medium, and high doses of corilagin from the Gorgon shell source in the experiment.

**Figure 7:**
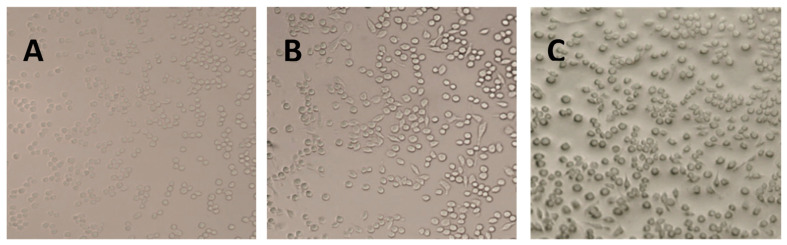
Morphology of Raw264.7 cells under different treatment conditions: (**A**) control cells, (**B**) lipopolysaccharide-stimulated cells, and (**C**) cells treated with corilagin from *Euryale ferox* Salisb shell.

Raw264.7 is a mononuclear macrophage derived from the leukemia virus in Balb/c mice [18]. The health of the cells can be impacted by the state of the cells, and since Raw264.7 cells are small and bright in appearance, they are not in a good shape. After the LPS stimulation, the cells formed a long shuttle with elongated false feet [19]. Additionally, pseudopodia were reduced in cells treated with corilagin, with most of the cells being round in shape (Figure 7). The results indicated that corilagin from *Euryale ferox* Salisb could mitigate inflammation by inhibiting the differentiation of Raw264.7 cells.

### 3.6. Effects of Corilagin on LPS-Induced NO Secretion by Raw264.7 Macrophages

**Figure 8:**
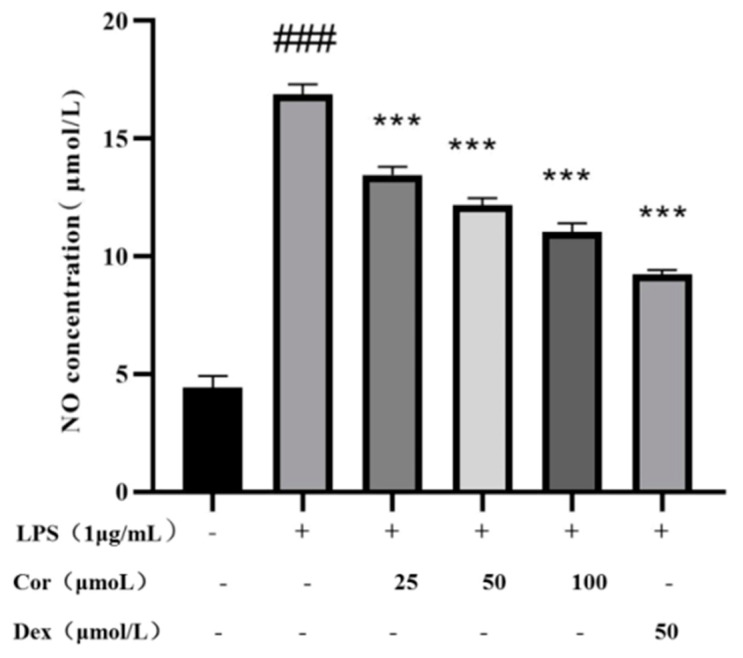
The effect of corilagin from *Euryale ferox* Salisb shell on NO secretion of Raw264.7 cells. (Compared with the control group, ### *p* < 0.001; Compared with LPS group, *** *p* < 0.001).

NO is an endogenous synthetic gas signal molecule, synthesized in the cytoplasm, which quickly diffuses through the cell membrane. The molecule rapidly reacts with other free radicals, producing high levels of active peroxidase (oxidant) and other active nitrogen derivatives. These molecules can reflect inflammation and diseases such as atherosclerosis [20]. The effects of NO secretion on the volume of Raw264.7 cells induced by LPS via the Griess method were investigated. The results (Figure 8) depict that at the concentration of higher than 25, 50, and 100 μmol/L, the corilagin intervention resulted in a significant reduction in the volume of LPS-induced Raw264.7 cells’ supernatant, indicating the potential inflammation-relieving activity of corilagin, which concerns its ability to suppress the release of NO.

### 3.7. The Effect of Corilagin on the Expression of Inflammatory Factors in LPS-Induced Raw264.7 Macrophages

**Figure 9:**
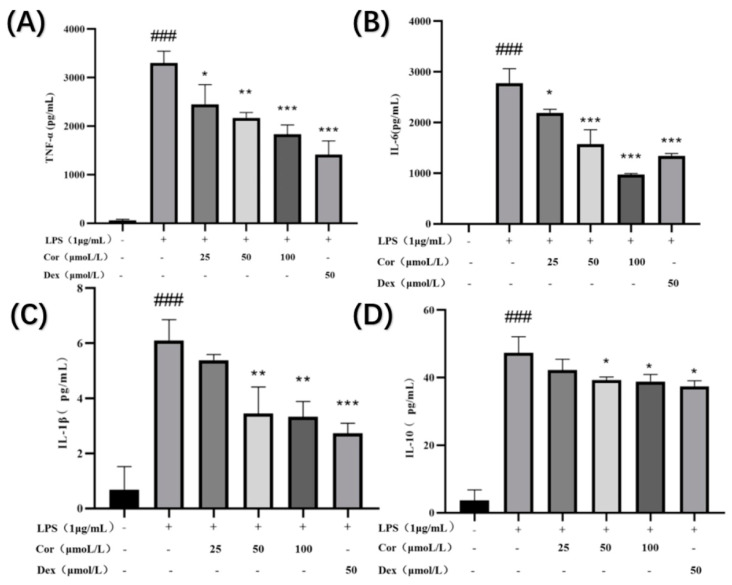
Effect of corilagin on TNF-α, IL-6, IL-1β, and IL-10 secretion of Raw264.7 cells: the amount of (**A**) TNF-α, (**B**) IL-6, (**C**) IL-1β, and (**D**) IL-10 secreted in the cell supernatant. (Compared with the control group, ### *p* < 0.001; Compared with LPS group, * *p* < 0.05, ** *p* < 0.01 and *** *p* < 0.001).

When macrophages are activated, inflammatory cytokines such as inflammatory enzymes and TNF-α, IL-6, IL-1β, and IL-10 are secreted. Following anti-inflammatory drug intervention, macrophages secrete anti-inflammatory factors, which are responsible for anti-inflammatory effects. The investigation of the effect of corilagin on the secretion of TNF-α, IL-6, IL-1β, and IL-10 in Raw264.7 cells (Figure 9) revealed that treatment with corilagin at 25, 50, and 100 μmol/ L significantly reduced the secretion of TNF-α and IL-6 in LPS-induced Raw264.7 cells. At 50 and 100 μmol/L, corilagin significantly reduced the secretion of IL-1β and IL-10 in LPS-induced Raw264.7 cells.

### 3.8. The Effect of Corilagin on the Content of Reactive Oxygen Species in Raw264.7 Cells

**Figure 10:**
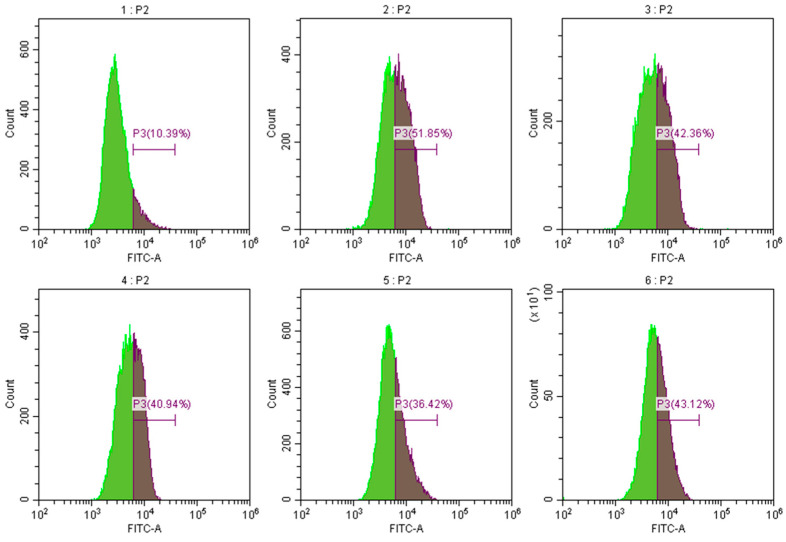
Effect of corilagin on ROS content of Raw264.7 cells. Pictures 1–6 correspond to the control group, LPS treatment group, 25, 50, 100 μmol/L of corilagin treatment group, and the 50 μmol/L dexamethasone treatment group, in which P3 represents intracellular reactive oxygen species content.

Activated oxygen is an active oxygen family, which includes superoxide and hydroxyl radicals that stimulate macrophages and neutrophils. This reactive oxygen species is produced by many biologically active media. Reactive oxygen species and other pro-inflammatory factors activate nuclear transcription factors (NF-κB) and cell nuclear binding, thereby promoting inflammatory factor transcription, sometimes resulting in inflammatory allergic reactions. These factors play a key role in the inflammation and wound healing processes, which lack specificity in bacteria. Excessive active oxygen can destroy the integrity of the mitochondrial membrane, resulting in changes in mitochondrial permeability; thus, eliminating tissue and damaging active oxygen is highly important. The effects of corilagin on the content of reactive oxygen species in Raw264.7 cells at 25, 50, 100, and 50 μmol/L of dexamethasone are presented in Figure 10. Following the intervention with corilagin at 25, 50, 100, and 50 μmol/L of dexamethasone, the intracellular ROS was reduced by 9.49%, 10.91%, 15.43%, and 8.73%, respectively. The results demonstrate that the corilagin from *Euryale ferox* Salisb shell can prevent the inflammatory reaction by reducing reactive oxygen species.

### 3.9. Effects of Corilagin on the Gene Expression of TNF-α, IL-6, iNOS, and COX-2 in LPS-Induced Raw264.7 Macrophages

**Figure 11:**
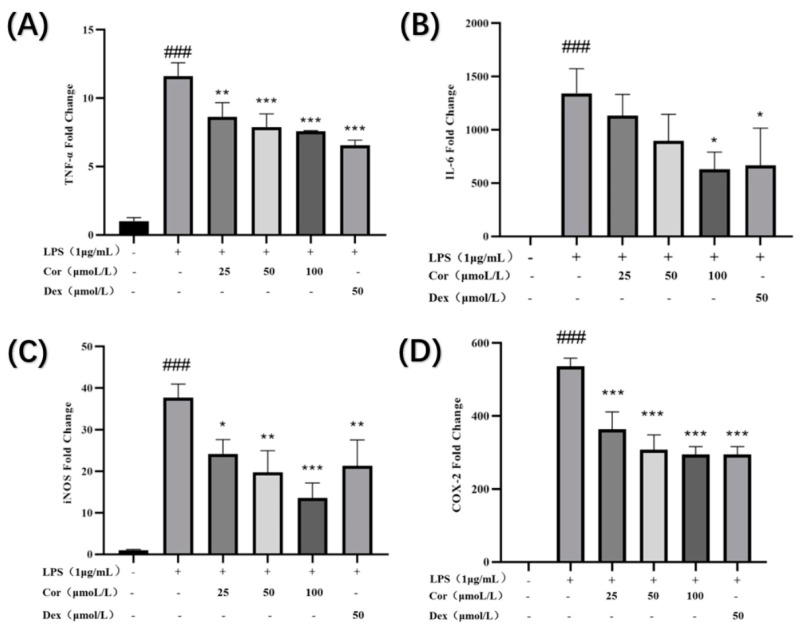
Effect of corilagin on TNF-α, IL-6, COX-2, and iNOS expression of Raw264.7 cells: expressions of (**A**) TNF-α, (**B**) IL-6, (**C**) iNOS, and (**D**) expression of COX-2. (Compared with the control group, ### *p* < 0.001; Compared with LPS group, * *p* < 0.05, ** *p* < 0.01 and *** *p* < 0.001).

The results (Figure 11) demonstrate that after the intervention of 25, 50, and 100 μmol/L derived from the Gorgon shell, the levels of gene expression of TNF-α, iNOS, and COX-2 were significantly reduced in a dose-dependent manner. Additionally, intervention by 100 μmol/L of corilagin significantly reduced the level of gene expression of IL-6. The nitric oxide synthase (NOS) can convert left-transverse arginine to left-transverse citrulline, thereby causing NO production, which can be classified into constitutive and inducible. Many studies have suggested that continuous and excessive NO production is primarily associated with the high expression level of inducible nitric oxide synthase (iNOS) [21]. The results indicate that the corilagin could reduce the secretion of NO by lowering the expression of iNOS, thereby exerting its anti-inflammatory effect. In addition, the level of COX-2 is increased in the inflammatory process, causing excessive production of PGE2, resulting in an excessive inflammatory response. The results also display that the corilagin could downregulate the expression of COX-2 to alleviate inflammation. TNF-α and IL-6 are the most common inflammatory cytokines, which play a vital role in inflammation. The results demonstrate that corilagin could reduce the gene expression of TNF-α and IL-6, thereby reducing the secretion of TNF-α and IL-6 and achieving an anti-inflammatory effect.

### 3.10. The Effect of Corilagin on the Expression of Key Proteins in the NF-κB Signaling Pathway in Raw264.7 Cells

**Figure 12:**
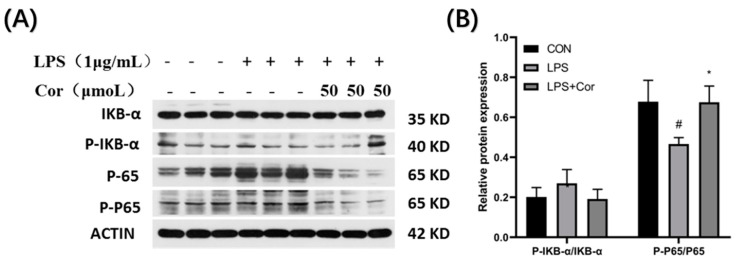
Effect of corilagin on the expression of NF-κB pathway-related proteins in Raw264.7 cells. (A) Western Blot results, (B) Relative protein expression (Compared with the control group, # *p* < 0.05; Compared with LPS group, * *p* < 0.05).

The effect of corilagin from the Gorgon shell on the expression of NF-κB pathway-related proteins in Raw264.7 cells is presented in Figure 12. The protein assay of IκB-α revealed that LPS stimulation and intervention by Gorgon shell-derived corilagin produced no significant effect on phosphorylation of IκB-α. Additionally, the phosphorylation of P65 was significantly downregulated following LPS stimulation, whereas the treatment with 50 μmol/L of corilagin resulted in upregulation of the phosphorylation of P65.

### 3.11. The Effect of Corilagin on the Expression of Key Proteins in the MAPK Signaling Pathway in Raw264.7 Cells

**Figure 13:**
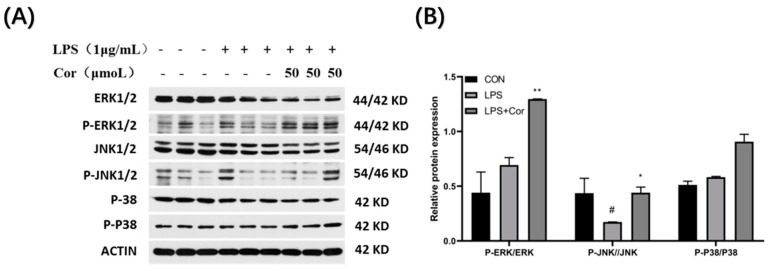
Effect of corilagin on the expression of MAPK pathway-related proteins in Raw264.7 cells. (A) Western Blot results, (B) Relative protein expression (Compared with the control group, # *p* < 0.05; Compared with LPS group, * *p* < 0.05 and ** *p* < 0.01).

The effect of corilagin from the Gorgon shell on the expression of MAPK pathway-related proteins in Raw264.7 cells is presented in Figure 13. The ERK protein assay revealed that LPS stimulation produced no significant effect on the phosphorylation of ERK, whereas the phosphorylation of ERK was significantly upregulated after the intervention of corilagin at 50 μmol/L. It was also found that the phosphorylation of JNK was significantly downregulated following LPS stimulation, while the intervention by 50 μmol/L of corilagin resulted in upregulation of phosphorylation of JNK. Additionally, both LPS stimulation and treatment with 50 μmol/L of corilagin produced no significant effect on the phosphorylation of P38.

### 3.12. The Effect of Corilagin on the Expression of Key Proteins in the PI3K-AKT Signaling Pathway in Raw264.7 Cells

**Figure 14:**
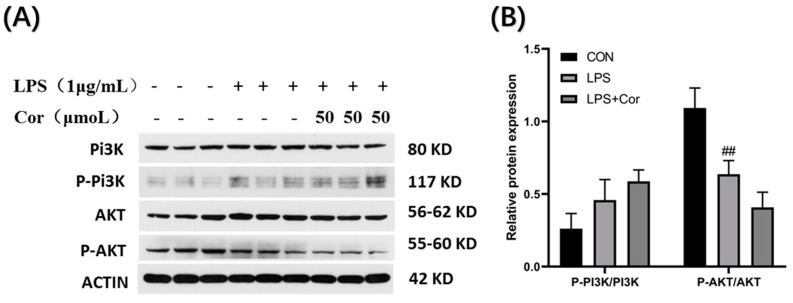
Effect of corilagin on the expression of PI3K-AKT pathway-related proteins in Raw264.7 cells. (**A**) Western Blot results, (**B**) Relative protein expression (Compared with the control group, ## *p* < 0.01).

The effect of corilagin from the Gorgon shell on the expression of PI3K-AKT pathway-related proteins in Raw264.7 cells is depicted in Figure 14. The PI3K protein assay revealed that both stimulations by LPS and intervention with 50 μmol/L of corilagin produced no significant effect on the phosphorylation of PI3K. Compared with the blank group, the phosphorylation of AKT protein was significantly downregulated following LPS stimulation, whereas the intervention with 50 μmol/L of corilagin produced no significant effect on the phosphorylation of AKT protein.

The investigation of the effect of the corilagin intervention revealed that no significant effect was produced on the expression of key proteins in the NF-κB signaling pathway in Raw264.7 cells. Similarly, stimulation by LPS produced no significant effect on the phosphorylation of IκB-α but significantly downregulated the phosphorylation of P65. In the MAPK signaling pathway, LPS stimulation had no significant effect on the phosphorylation of ERK and P38 but significantly inhibited the phosphorylation of JNK. In addition, in the PI3K-AKT signaling pathway, LPS stimulation had no significant effect on the phosphorylation of PI3K but significantly downregulated the phosphorylation of AKT. Previous studies have demonstrated that when macrophages develop lipopolysaccharide tolerance, LPS stimulation results in reduced P65 phosphorylation in the NF-κB signaling pathway, as well as that of ERK, JNK, and P38 in the MAPK signaling pathway [21]. The results from Western blot analysis demonstrate that the macrophages were lipopolysaccharide-tolerant and that LPS at 50 μmol/L was tolerated in this study. Additionally, the intervention of corilagin originated from the Gorgon husk significantly upregulated the phosphorylation of P65 and JNK proteins and relieved the lipopolysaccharide tolerance state of Raw264.7 cells, indicating its potential immunoregulatory role [21].

## 4. Discussion

Corilagin is a polyphenolic tannin compound [16], which is widely found in geranium, bead, white clover, longan, *Phyllanthi fructus*, and other plants [22]. Structurally, ellagic acid is a dilactone of hexahydroxy biphenyl acid (HHDP). Corilagin, a kind of ellagitannin which is a condensation of ellagic acid, has good antioxidant potential. After optimizing the extraction process of ellagitannin, Anindita Paul et al. obtained the result that ellagitannin could combine well with catalase through calculation and analysis [23]. Studies have demonstrated that corilagin exhibits antitumor, antiviral, and antibacterial activities in addition to anti-inflammatory and antioxidant effects, suggesting its potential use as an agent in the preventive treatments of cardiovascular diseases [13,22,24,25,26]. Corilagin sourced from Gorgon husk was extracted and identified during the early stages of a study on Gorilla husk by our research team, but its anti-inflammatory properties have not yet been revealed.

Cyber-pharmacology efficiently integrates research content, utilizing high-throughput computing methods and software. By setting different screening conditions, proteins interacting with small molecules can be accurately predicted, to predict protein-related metabolic pathways [27]. In this study, we identified 307 targets of corilagin using PharmMapper. The subsequent GO and KEGG enrichment analyses revealed that the anti-inflammatory effects of corilagin are primarily associated with MAPK and TOLL-like receptor signaling pathways. Additionally, LPS and other pro-inflammatory factors can induce the phosphorylation of MAPK signaling pathway-associated proteins and trigger the expression of iNOS genes in the nucleus.

Findings from this study demonstrate that corilagin, a Gorgon fruit source, could significantly inhibit the production of reactive oxygen species induced by LPS in macrophages, as well as the related oxidative stress responses in cells. These results further confirm the findings reported in previous studies regarding the positive correlation between the degree of oxidative stress and the degree of inflammation [28] and that inhibiting excessive production of reactive oxygen species can suppress the inflammatory response. Analysis of NO secretion revealed that the intervention of corilagin from Gorgon shell significantly reduced NO content in the supernatant of LPS-induced Raw264.7 cells.

Analysis of the gene expression of inflammatory factors demonstrated that the corilagin intervention significantly reduced the expression of IL-6 and TNF-α genes compared with LPS-induced macrophage inflammation. Corilagin also significantly downregulated the expression of iNOScox-2, and nitric oxide synthase (NOS) could convert L-arginine to L-citrulline, thereby causing the release of NO. Numerous studies have suggested that persistent and excessive NO production is mainly attributed to the overexpression of inducible nitric oxide synthase (iNOS) [29]. In addition, the expression of COX-2 is elevated during the inflammation process, causing the overproduction of PGE2, which results in an excessive response to inflammation [30]. This proves that corilagin, a source of a gorgonian shell, could reduce the secretion of NO by downregulating the expression of iNOS, thereby exerting its anti-inflammatory effect.

Many physiological and pathological responses in mammalian cells and tissues are mediated by MAPK signaling, including stress responses, inflammation, and apoptosis. Phosphorylation of ERK1/2 and p38 promotes the production of inflammatory factors, including TNFα, IL-6, and IL-8 [31]. In addition, TNF-α also stimulates the MAPK cascade and promotes IL-8 secretion [32]. The analysis conducted in this study revealed that phosphorylation levels of ERK and JNK proteins increased following treatment with corilagin.

Related studies have reported that p38 of MAPK and PI3K-Akt can regulate LPS-induced gene expression by controlling the hyperphosphorylation and nuclear translocation of p65 of NF-κB [33]. Corilagin could also significantly upregulate the phosphorylation of P65 protein. This study also examined the expression of related proteins in the PI3K-Akt signaling pathway, which revealed that LPS induction significantly reduced the phosphorylation level of AKT.

Previous studies have demonstrated that when macrophages develop lipopolysaccharide tolerance, LPS stimulation can reduce the phosphorylation of P65 protein in the NF-κB signaling pathway, as well as that of ERK, JNK, and P38 in the MAPK signaling pathway [34]. Western blot analysis demonstrated that the intervention of 50 μmol/L of gorgonian shell-derived corilagin could significantly upregulate the phosphorylation of P65 and JNK, relieving the lipopolysaccharide tolerance state of Raw264.7 cells, thereby exerting an immune-regulating effect. The results indicate that macrophages developed lipopolysaccharide tolerance and that corilagin interfered with the phosphorylation of P65 and JNK proteins and relieved the macrophages of their tolerance. Notably, lipopolysaccharide tolerance plays an immunomodulatory role [24].

## Figures and Tables

**Table 1:** Primer sequences.

Gene Name	Forward Primer (5→3)	Reverse Primer (3→5)
*β-actin*	CTACCTCATGAAGATCCTGACC	CACAGCTTCTCTTTGATGTCAC
*TNF-α*	ATGTCTCAGCCTCTTCTCATTC	GCTTGTCACTCGAATTTTGAGA
*IL-6*	CTCCCAACAGACCTGTCTATAC	CCATTGCACAACTCTTTTCTCA
*COX-2*	ATTCCAAACCAGCAGACTCATA	ATTCCAAACCAGCAGACTCATA
*iNOS*	CGGACGAGACGGATAGGCAGAG	GGAAGGCAGCGGGCACATG

## Data Availability

The datasets generated for this study are available on request to the corresponding author.

## Supplementary Materials

The following are available online at https://www.mdpi.com/article/10.3390/foods12050979/s1, Figure S1. Mass spectra of corilagin; Figure S2. Claritin extraction process.
