# Supplementary material for: The Effect and Mechanism of Corilagin from Euryale Ferox Salisb Shell on LPS-Induced Inflammation in Raw264.7 Cells
Source: Foods. 2023 Feb 25;12(5):979. doi: 10.3390/foods12050979 (PMC10000429; doi:10.3390/foods12050979)
Supplement: Supplementary file 1 [file foods-12-00979-s001.zip › foods-2173433-SI.pdf]

The effect and mechanism of Corilagin from *Euryale ferox* Salisb shell on LPS-induced inflammation in Raw264.7 cell

Supplementary Materials

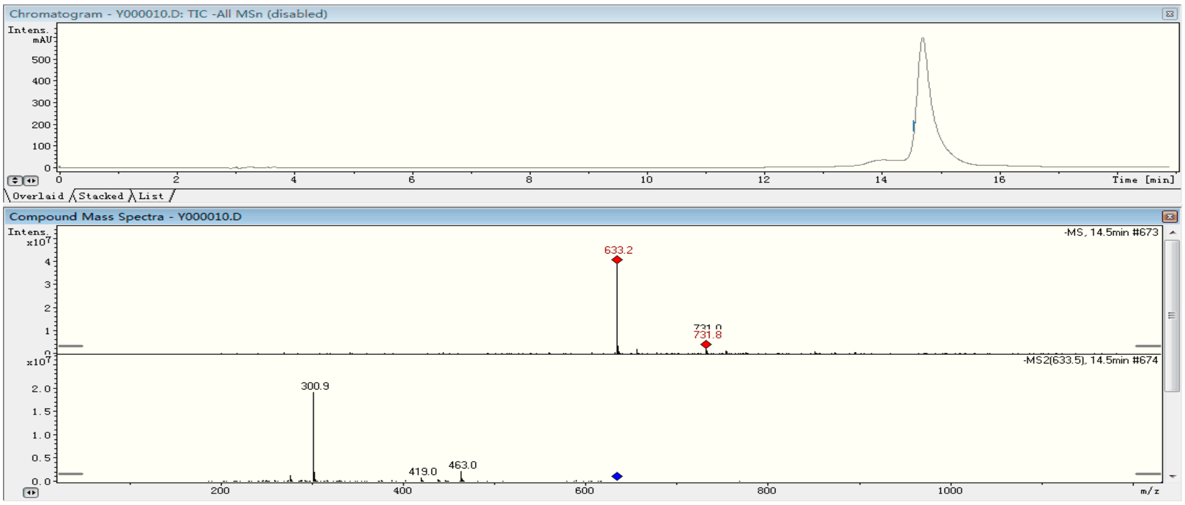

Figure S1. Mass spectra of corilagin.

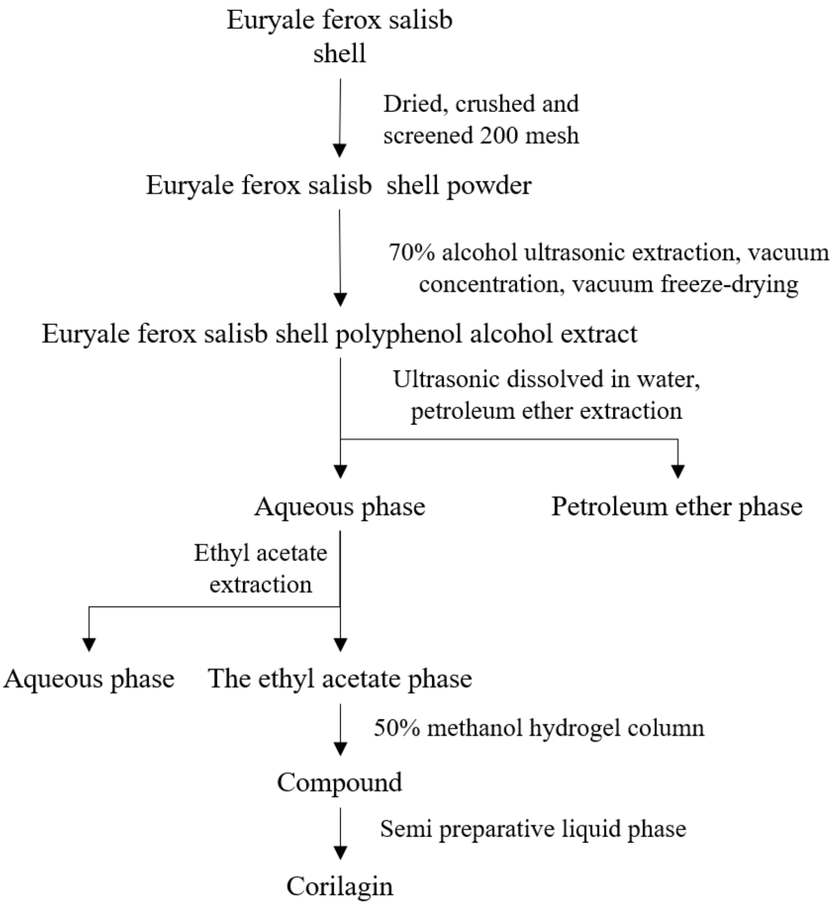

Figure S2. Claritin extraction process.
